# The impact of economic uncertainty on bank efficiency—the moderating role of country governance

**DOI:** 10.1016/j.heliyon.2024.e27905

**Published:** 2024-03-11

**Authors:** Heng Luo, Fakarudin Kamarudin, Normaziah Mohd Nor

**Affiliations:** a School of Business & Economics, Universiti Putra Malaysia, Serdang, Malaysia; b School of Digital Economy and Industry, Jiangxi University of Engineering, Xinyu, Jiangxi, China

**Keywords:** Economic uncertainty, DEA, Conventional banks, Islamic banks, Country governance

## Abstract

This study examined the impact of economic uncertainty (EU) on Islamic banks (IBs) and conventional banks' (CBs) efficiency in countries that meet a standard where 1% share of Islamic banking assets is part of their total domestic banking sector assets. In addition, this study explored the moderating effect of country governance (CG) by employing the quantitative methodology based on secondary data from 2006 to 2021. The data analysis was done through ordinary least square, fixed effect model, and the random effect model. EU was found to enhance bank efficiency based on the basic regression results. CG moderated the positive effect of EU on bank efficiency. Additional robustness tests showed that EU was positively related to both types of banks' efficiency. The value of the paper is unique in that few papers have investigated the moderating effect of CG on the impact of EU on banks' efficiency, which enhances comprehension of EU and CG. These results highlight important policy implications whereby banks should continue to invest in and improve their risk management strategies. In addition, governments and regulatory bodies should prioritise good governance practices as these can improve banks’ efficiency.

## 1 Introduction

### 1.1 Background

In 2021, the global economy witnessed a growth of 5.9% compared to a contraction of −3.1% as recorded in 2020 due to the COVID-19 pandemic based on the Islamic Financial Services Industry (IFSI) Stability Report 2022 (https://www.ifsb.org/sec03.php). The Islamic financial market was also anticipated to arrive at USD 3.06 trillion by the end of 2021, representing an increase of 11.3% in assets compared to the year over year comparison in US dollars.

The boom in Islamic finance has attracted the attention of various stakeholders, such as policymakers and researchers, who have concentrated on this special banking system and paid attention to the gap between conventional banks (CBs) and Islamic banks (IBs). In fact, there is now a thriving literature that studies whether IBs outperform CBs [[1], [2], [3], [4], [5]]. Not only that, economic uncertainty (EU) has also drawn a lot of attention from researchers [], where the level of EU can be measured by counting how frequently the word “uncertainty” appears in reports. All this shows how imperative it is to analyse and understand the dynamics of the recovery of the global economy, including the role played by the IFSI.

### 1.2 Problem statement and motives

The global financial landscape has witnessed significant fluctuations and uncertainties over the past decades, especially after the 2008 financial crisis, where businesses, households, and policymakers have been influenced by uncertainty [6]. A violated policy environment, for instance, can make it harder to schedule financial activities, resulting in uncertainty in many aspects of financial life. When EU develops, investors can put off making investments until they have more information [7]. Hence, higher uncertainty leads to decreased credit supply, downgraded consumption, ebbs in investment booms, and increased problematic loans.

Due to the possibility of spillovers and contagion effects, economic policy uncertainty that originates from specific nations or industries can impact entire industries such as banks, which then influence their efficiency. However, these banks can innovate and adapt their business models, offer new products or services with robust risk management practices, and implement risk management to improve their performance in an environment of high uncertainty. Hence, based on the above discussion, this study sought to understand whether EU affects IBs and CBs in both the normal setting and in times of crisis. More importantly, no research has been done to investigate whether country governance (CG) affects the relationship between EU and bank efficiency. Thus, this research gap was addressed in this study by examining the influence of CG on the nexus of the EU and the efficiency of IB and CB's.

In extant literature, there are two main EU indexes, namely the economic policy uncertainty index of [8] and the World Economic Uncertainty Index (WUI) of [9]. The former measures the frequency of specific words appearing in articles from newspapers using a text-based uncertainty method. The latter is constructed by tallying the number of times the term "uncertainty" (or a synonym) appears in reports from the Economist Intelligence Unit (EIU). To examine the impact of the EU on the two types of banks’ efficiency in this study, the WUI developed by Ref. [9] was chosen as the proxy of EU as it is more standardised, is constructed from extremely high accurate EIU, and covers more countries [10].

### 1.3 Contribution

There are three major innovations of this work compared to extant research. First, recent literature has focused on the EU and stability nexus [[10], [11], [12]] as well as the performance or profitability nexus [13,14]. Other studies have examined the impact of EU on banks’ behaviour, such as lending behaviour [15], decreases in bank credit supply [16], and negative credit growth [17]. This study adds to the body of existing knowledge by examining the role of CG in moderating the nexus between EU and bank efficiency.

Second, this paper contributes to the banking system literature. This research adds to the body of knowledge already available on bank efficiency by being the first to investigate the influence of EU on CBs and IBs' efficiency in countries that meet a standard where 1% share of IB assets is in their total domestic banking sector assets. In preceding studies, the influence of EU on IBs had not been the primary focus; instead, scholars had concentrated on CBs in economically powerful countries such as Europe [12], the US [16], and China [18]. Nevertheless, the legal and regulatory environment in the sample countries of this country was designed to accommodate to the principles and practices of Islamic finance and the financial activities and products offered by these countries' banks that adhere to Shariah principles. Moreover, there were significant differences in economic and religious policies between the sample countries in this study and these countries. As such, the insights drawn from earlier research conducted in other areas might not apply to the areas this study chose to focus on. By incorporating governance as a moderating factor, this study adds to the body of knowledge about the banking system's moderating variables.

Third, this paper enriches the body of knowledge as it explored the key factors affecting the efficiency of CBs and IBs. Many research works have looked into several aspects that will affect banks' efficiency, such as CG [4], Shariah supervision [19], and globalisation [20], however, this study revealed that EU is another important macro determinant.

### 1.4 Structure of the paper

The rest of the paper is structured as follows. Section 2 is the literature review. Section 3 describes the data and methodology. Section 4 reports the empirical results. The final section highlights the main findings, conclusions, and implications.

## 2 Literature review

### 2.1 The relationship between economic uncertainty and banks

The transaction cost theory [21] suggests that transaction costs rise with high uncertainty because businesses have to spend more money to protect themselves against unanticipated hazards. However, increased cost is accompanied by lower efficiency. Thus, EU depresses efficiency. In the Adaptive Management Theory [22], organisations can use an adaptive management strategy in unpredictable environments. For banks, this means that they can proactively modify their risk management techniques in response to evolving circumstances. Hence, EU expedites managerial learning and enhances the efficiency of banks.

In the empirical literature on EU, the influence of the EU on the economy has received scant attention. For instance, EU in the United States and Europe was found to impair the development of the economy. In addition, uncertainty also heavily affects volatilities, investment rates, and employment growth [8]. Uncertainty reduces the number of mergers and acquisitions (M&A) and increases the energy devoted into M&A [23]. It interferes with the growth of the economy by reducing corporate investment due to the uncertain outcome of investment and the higher cost of external lending [24]. It also fosters technological innovation in developed regions and depresses innovation in developing areas [25]. As a result, investors become negative and stocks’ performance had to keep pace with the attitude of investors during the period of high EU [26]. Foreign direct investment is also depressed by EU [27].

Some studies in the EU literature have linked the behaviour of banks to EU. A higher level of uncertainty has been found to be accompanied by prudential banking behaviour together with a conservative credit quota [28]. In addition, EU has been found to have a positive effect on bank stock returns in the Middle East and North Africa (MENA) region [29] and depresses the lending behaviour of banks [15]. Loan loss provisioning has also been found to be positively connected to the level of uncertainty in US banking [30]. Increases in EU are also associated on average with less earnings management among Japanese banks and this effect is weakened with firms with a main bank [31]. EU has also been found to suppress bank liquidity creation and decrease the supplementation of credit [32]. However, all in all, these studies have not extended their analysis to examine how changes in bank activities affect bank efficiency in facing EU.

Only a limited number of studies have explored the relationship between EU and banks. Existing research have focused primarily on the impact of EU on certain financial indicators and the stability of banks. However, it had consistently yielded negative correlations. Very few studies have delved into studying bank efficiency. For instance, there is a negative correlation between the EU and some measurements of bank profitability and this effect is more pronounced in some regions [14]. EU has also been found to be negatively and positively correlated with financial stability (proxied by the Z-score) and stability (measured by non-performing loans) [11]. This has undermined the stability of European banks that may result from declining economic environment [12]. EU has also been found to be negatively correlated with banks' gross non-interest income and expense [33]. However, all in all, the above studies did not study the nexus between efficiency and EU. Hence, hypothesis was developed.

Hypothesis 1 EU and the efficiency of both kinds of banks are favourably associated.

### 2.2 The influence of country governance on the nexus between economic uncertainty and the efficiency of the two types of banks

Researchers have seldom investigated the moderating role of CG on the impact of EU on bank efficiency in the existing body of literature. However, some studies have found that good governance can moderate the effect of the EU on financial institutions. For instance, governance has been found to moderate the impact of EU on stability, and this moderation effect varies across regions [11]. In another study [15], investigated the EU and bank lending nexus combined with institutional quality, and the results indicated that high-quality institutions had a substantial role in increasing loan supply and reducing the negative consequences of uncertainty. In addition, CG was found to influence the efficiency of banks. Regulatory quality can also increase the efficiency of banks as the professional handling of bureaucracy and political stability can impede the development of banks, where excessive political stability could be detrimental for better bank performance [4].

Hypothesis 2 CG can moderate the relationship between the EU and both types of banks' efficiency.

## 3 Data and methodology

To analyse the influence of EU on the efficiency of CBs and IBs, data was acquired from multiple sources. Firstly, bank information was extracted from Bankfocus between 2006 and 2021 in countries that met the criteria where the share of Islamic banking assets in their total domestic banking sector assets was over 1%. All data was supplemented by a linear interpolation method. The reason why the period of 2006–2021 was chosen was that a lot of data about the bank is abundant since 2006. Hence, all banks whose main variables for calculating the data envelopment analysis (DEA) score were not available were not included.

Second, to measure the level of the EU, the WUI was utilised, as well as data from Ref. [9] who constructed the index using text analysis.

Third, macroeconomic data including inflation and gross domestic product (GDP) were collected from the WDI.

Fourth, CG was gathered from the Worldwide Governance Indicators (WGI). For this indicator, an improved degree of governance is indicated by a higher value.

Based on the data availability, the final sample included 14 countries, comprising a total of 3980 observations for CBs and 1228 observations for IBs.

### 3.1 Dependent variable: efficiency

The variables selected for the DEA model were based on prior research. Banks play a major role in both lenders and borrowers, hence the following were used: (i) deposits and short-term funding and (ii) fixed assets as inputs to generate (i) loans and (ii) total financial assets, including securities, utilising the existing methods of [3,[34], [35], [36], [37], [38]]. The details for variables used to calculate the DEA score are outlined in Table 1.

**Table 1:** DEA inputs and outputs.

	Variables	Definition
Inputs		
X1	Deposits	Deposits & short-term funding
X2	Physical capital	Fixed Assets
Outputs		
Y1	Loan	Gross loans
Y2	Investment	Total financial assets: securities

### 3.2 Independent variable: economic uncertainty

WUI was chosen as the proxy for EU. This index was constructed by counting the number of times the word "uncertainty" (or its variant) appeared in the EIU country reports. A lower value represents a more stable economic environment. The data is available at https://worlduncertaintyindex.com/data/. The methodology of [10,29] was followed, where the simple average of the four quarters was taken to formulate a yearly index. For the robust test, the method of [10] was followed by taking the average of the first two quarters of WUI to replace the independent variable.

### 3.3 Moderating variable: country governance

This indicator is reported for numerous countries by the WGI project.

Following previous studies of [11,15,32] who considered CG as a moderator when examining the relationship between EU and the characteristic of banks, this index was chosen as the moderating variable to analyse the nexus between uncertainty and bank efficiency. Additionally, the methodology employed by Ref. [39] was followed in computing the CG score by averaging the values of the six dimensions of institutional quality.

### 3.4 Control variable

Three bank-level variables were considered, which included bank size, credit risk, and capitalisation. Following previous studies of [20,36,[40], [41], [42]], the natural logarithm of total assets was chosen as the proxy of bank size. The efficiency of a bank was supposed to be positively correlated with its size as large banks are able to benefit from economies of scale. Credit risk was measured by incorporating the ratio of loan loss provision to gross loans [20,43]. This ratio was also a proxy for the quality of the loan. Credit risk was expected to be negatively related to a bank's efficiency. Equity over total assets was a proxy for capitalisation [37,44]. It was anticipated that capitalisation would have a positive relationship with a bank's efficiency because firms with high equity-to-capital ratio have a lower likelihood of insolvency.

With regard to country-level variables, the method of [35] was followed in choosing GDP and inflation. Hence, the larger the GDP level, the more efficient the banks were. Due to information asymmetry and disparities in the accuracy of consumer price index (CPI) expectations, the interest rates of banks were adjusted and revenue was gained in the prediction [20]. Hence, the sign of GDP and inflation were anticipated to be positive.

The definition and sources of variables are described in Table 2.

**Table 2:** Variables explanations.

	Variable	Symbol	Description	source
Dependent variable	Efficiency score	Te	Measure the efficiency of the bank(log).	Calculated by the author
Independent variable	Economic uncertainty	Wui	Measure the level of uncertainty	(http://www.policyuncertainty.com/)
Bank-level control variables	Size	InAsset	Bank's total assets (log).	BankFocus
	Credit risk	quality	Loan Loss Res./Gross Loans	BankFocus
	Capitalization	capital	Equity/Total assets	BankFocus
Country-level variables	GDP	Ingdp	GDP (constant 2015 US$)(log).	World Bank World Development Indicators
	CPI	Incpi	Consumer price index (2010 = 100) (log).	World Bank World Development Indicators
Moderating variable	country governance	CG	Average of six dimensions	World Banks Governance Indicators(http://info.worldbank.org/governance/wgi/#reports)

### 3.5 Assessing efficiency

The DEA method was employed in the first stage as it is widely used and accepted. In addition, this method requires no parametric specification determination [45]. [46] proposed the DEA method based on previous research findings. Their findings solved the difficulty to evaluate the relative efficiency of multiple investments and products. In a set of comparable decision-making units (DMUs), this method can determine the DMUs that show the best practice to form an effective frontier. The ratio of output is the relative efficiency of the structure (input) output, also known as technical efficiency (TE).

DMUs are supposed to be (k = 1, …, K) with the vector of input indicated as x = (x_1_, …, x_N_) ∈ℜ^*N*+^ and the vector of output represented as y = (y_1_, …, y_M_) ∈ℜ^*M*+^. The efficiency of the DMU can be calculated using Equation (1):

(1) $${TE}_{K}=\frac{\alpha_{1}y_{1k}{+\alpha }_{2}y_{2k}+{\cdots \alpha }_{M}y_{Mk}}{\beta_{1}x_{1k}{+\beta }_{2}x_{2k}+{\cdots \beta }_{N}x_{Nk}}=\frac{\sum_{m=1}^{M}\alpha_{m}y_{mk}}{\sum_{n=1}^{N}\beta_{n}x_{nk}}$$

Where:

TE_K_ = the technical efficiency score given to the K-th DMU;

α = output weights;

β = input weights.

### 3.6 Second stage analysis

This study used parametric (*t*-test) and nonparametric (Mann-Whitney [Wilcoxon] and Kruskal–Wallis) tests to examine the differences in the efficiency score of the IBs and CBs. Then, the model in section 3.7 was used to reveal factors that may influence banks' efficiency.

### 3.7 Econometric model

Following the method of [47,48] this study employed the ordinary least square (OLS), fixed effect model (FEM) and random effect model (REM) to thoroughly examine the relationship between EU and banks’ efficiency. The Breusch Pagan and Lagrangian Multiplier (BP and LM) tests were the prerequisite stage since they might determine whether panel or pooled data was suitable. If the p-value of these tests was significant at 5% level, panel data was chosen. The Hausman test was used to determine FEM or REM.

Thus equations (2), (3)) are proposed:

(2) $${Te}_{it}=\alpha_{0}+\alpha_{1}{Wui}_{jt}+\sum_{a=1}^{3}\beta_{a}{BC}_{it}+\sum_{b=1}^{2}{\delta_{b}CC}_{jt}+\varepsilon_{ijt}$$

(3) $${Te}_{it}=\alpha_{0}+\alpha_{1}{Wui}_{jt}+\alpha_{2}*{\text{CG}}_{\text{jt}+}\sum_{a=1}^{3}\beta_{a}{BC}_{it}+\sum_{b=1}^{2}{\delta_{b}CC}_{jt}+\alpha_{3}{Wui}_{jt}*{CG}_{jt}+\varepsilon_{ijt}$$

where:

Te_it_ = efficiency of bank i at time t (log).

Wui_jt_ = Economic uncertainty of country j at time t.

CG_jt_ = Country governance of country j at time t.

BC_it_ = bank-level control variable of bank i at time t.

CC_jt_ = country-level control variable of country j at time t

ƹ_ijt_ = the error term.

## 4 Results

### 4.1 Univariate test

A univariate test was performed in all regions and sub-regions. In Table 3, three different tests can be seen in panel A, B, and C. The *t*-test results indicated that the mean of IBs was a little higher than that of CBs (0.129 > 0.111), however, results from the non-parametric Mann-Whitney (Wilcoxon) and Kruskal-Wallis tests had the opposite conclusion.

**Table 3:** Univariate test of efficiency on islamic vs. Conventional banks.

Region	No. Obs.	Panel A: *t*-test		Panel B: M-W		Panel C: K–W	
				Wilcoxon Rank-Sum test		Equality of Populations test	
		t (Prb > t)		z(Prb > z)		X^2^ (Prb > X^2^)	
**ALL**	5208	Mean	t	Mean Rank	z	Mean Rank	X^2^
CB	3980	0.111	−3.135***	2647.20	−3.689***	2647.20	13.611***
IB	1228	0.129		2466.12		2466.12	
**SA**	1104						
CB	905	0.122	−2.162***	570.19	−3.932***	570.19	15.459***
IB	199	0.165		472.04		472.04	
**MENA**	1378						
CB	969	0.100	−1.351***	718.86	−4.215***	718.86	17.776***
IB	409	0.115		619.95		619.95	
**EAP**	1932						
CB	1570	0.113	−6.506***	907.08	−9.749***	907.08	95.047***
IB	362	0.176		1224.19		1224.19	
**SSA**	794						
CB	536	0.103	6.443***	445.66	−8.528***	445.66	72.729***
IB	258	0.060		297.45		297.45	

Standard errors in parentheses.

*p < 0.1, **p < 0.05, ***p < 0.01.

### 4.2 Descriptive statistics

The details of variables are described in Table 4.

**Table 4:** Descriptive statistics for all banks.

variable	N	mean	sd	min	max
Inte	5208	−2.945	1.724	−15.41	0
InAsset	5208	14.26	1.724	7.983	19.52
CPI	5208	4.869	0.447	4.098	9.696
GDP	5208	26.04	1.070	23.28	27.70
quality	5208	5.920	14.63	−38.29	501.6
capital	5208	17.48	16.09	0.0750	104.7
wui	5208	0.153	0.137	0	0.938
CG	5208	−0.446	0.566	−1.728	0.721

The dependent variable had an average logarithmic value of −2.945 with a standard deviation of 1.724. Meanwhile, the average of EU in the sample was 0.153 with a standard deviation of 0.137. Regarding control variables, notable variations can be seen around the sample mean.

Table 5 presents the descriptive statistics of the CBs and IBs variables in the sample.

**Table 5:** Descriptive statistics for CBs and IBs.

	CB	IB
N	3980	1228
Inte	−2.874	−3.175
InAsset	14.35	13.99
CPI	4.823	5.02
GDP	26.09	25.85
quality	5.516	7.229
capital	15.98	22.34
wui	0.156	0.143
CG	−0.419	−0.533

In terms of bank size, it can be observed from Table 5 that CBs had on average a bigger size than IBs. CBs has a longer history and has a larger bank asset. In addition, IBs enjoy a higher equity-to-asset ratio than CBs as expected because IBs rely less on debt financing and more on equity-based funding from shareholders and investors. IBs have a higher credit risk level than CBs, which can be explained by the fact that IBs predominantly engage in asset-backed financing, which leads to a cautious approach in provisioning for potential loan losses.

Table 6 shows Pearson's correlation matrix which displays coefficients between variables. The highest correlation between the explanatory variables was below 0.6, which confirmed the absence of multicollinearity in the regression estimation. Multicollinearity exists in a multivariate study if the correlation coefficients with the explanatory variables were more than 0.8 [49]. The variance inflation factor (VIF) values reported in Table 7 indicated that there was no significant multicollinearity issue provided that the maximum VIF value was 1.52.

**Table 6:** Correlation matrix for the explanatory variable.

	Inte	InAsset	CPI	GDP	quality	capital	wui	CG
Inte	1							
InAsset	0.175***	1						
CPI	−0.228***	−0.150***	1					
GDP	−0.004	0.072***	0.004	1				
quality	−0.101***	−0.115***	0.056***	−0.117***	1			
capital	−0.144***	−0.489***	0.003	−0.017	0.171***	1		
wui	0.013	−0.141***	0.130***	−0.235***	0.023*	−0.019	1	
CG	0.195***	0.419***	−0.299***	0.109***	−0.198***	−0.295***	−0.059***	1

**Table 7:** VIF.

Variable	VIF	1/VIF
InAsset	1.52	0.659
capital	1.38	0.724
CG	1.37	0.73
CPI	1.13	0.883
wui	1.1	0.907
GDP	1.08	0.922
quality	1.07	0.937
Mean/VIF	1.24	

### 4.3 Basic results

In Table 8, control variables were included, namely the size of bank (lnAsset), credit risk (quality), capitalisation levels (capital), the log of GDP (Ingdp), and inflation (cpi). EU was also included in the model. The results from Table 8 indicated that the FEM was most suitable to be used in this study as the p value of the BP test and the Chi-square of the LM test was significant at 1% level or lower and the p value of the Hausman test was significant at 1% level or lower.

**Table 8:** Basic regression.

	CB			IB		
	ols	fe	re	ols	fe	re
constant	2.295***	54.856***	6.792***	−5.460***	45.202***	2.302
	(0.73)	(5.07)	(1.75)	(2.03)	(8.85)	(3.71)
InAsset	0.118***	0.179***	0.156***	0.057	−0.006	−0.039
	(0.02)	(0.02)	(0.02)	(0.04)	(0.05)	(0.05)
CPI	−1.476***	−0.197	−1.600***	−0.541***	−0.451***	−0.506***
	(0.10)	(0.18)	(0.11)	(0.08)	(0.08)	(0.08)
GDP	0.010	−2.291***	−0.181**	0.176**	−1.796***	−0.108
	(0.02)	(0.22)	(0.08)	(0.07)	(0.35)	(0.15)
quality	−0.001	0.007***	0.003	−0.012***	−0.007***	−0.008***
	(0.00)	(0.00)	(0.00)	(0.00)	(0.00)	(0.00)
capital	−0.011***	0.009***	0.003	−0.007**	0.016***	0.011***
	(0.00)	(0.00)	(0.00)	(0.00)	(0.00)	(0.00)
wui	1.099***	1.532***	1.484***	−0.776	0.574	−0.140
	(0.18)	(0.21)	(0.21)	(0.49)	(0.53)	(0.51)
N	3980	3980	3980	1228	1228	1228
r2	0.094	0.117		0.103	0.087	
r2_a	0.093	0.113		0.099	0.072	
F	68.884***	87.356***		23.469***	19.143***	
BP LM	chibar2(01) = 578.01***			chibar2(01) = 1242.32***		
chi2			410.981***			77.219***
Hausman		chi2(6) = 133.34***			chi2(6) = 58.66***	

Standard errors in parentheses.

*p < 0.1, **p < 0.05, ***p < 0.01.

The FEM in Table 8 showed that EU had a positive effect on the efficiency of CBs and IBs but was only significant for CBs. In addition, the coefficient of CBs was greater than IBs. In terms of the insignificant impact of EU on IBs, this can be explained by different operational foundations. IBs are characterised by an asset-backed nature, while CBs rely on an interest-based model. If the real economy suffers a disaster, Islamic finance will be influenced due to this dependence [50].

The positive sign in CBs may result from the effective anti-risk management of banks. During periods of EU, banks typically face higher levels of risk due to increased market volatility and unpredictability. In response, banks may enhance their risk management practices and focus on improving efficiency to offset various unknown results of the future. The outcomes of future EU can increase non-performing loans, unexpected withdrawal of money, and declined asset values. Hence, when faced with the above outcomes, it is important to implement stricter credit assessment processes, improve risk models, and increase loan prices to navigate uncertain economic conditions effectively.

The empirical results based on 14 countries for a period of 16 years showed that the higher the EU, the higher the loan price on average [51]. This positive relationship is consistent with the studies of [[52], [53], [54]]. In terms of the greater coefficient of CBs, CBs enjoy a longer history than IBs, and the experience and risk-management tools are more than IBs. This means that it is easier for CBs to operate in an environment of high uncertainty.

On a separate note, IBs offer ownership-based financing like Mudarabah and Musharakah as well as asset-based financing like Murabahah, which may be less attractive to customers, as they distribute risk and customers gain low payback. However, CBs provide customers with inflation-adjusted products, which adjusts with inflation and may be perceived to be better investment choices. This explanation is consistent with [14], who concluded that increases in economic policy uncertainty are associated on average with increases in bank profitability in Asia due to the distinct banking structure or regulation. Finally, Shariah supervisory boards (SSBs) may be a plausible explanation for the phenomenon because SSBs guide IBs and ensure compliance with Shariah principles, which can significantly back the credibility of IBs' products and influence customers' perception and confidence. The positive relationship between SSBs and IBs’ profitability is supported by the results of [5].

With regards to the control variables, the results indicated that increases in bank size were solely associated with an increase in CBs' efficiency. The coefficient of capitalisation was significantly and positively correlated with both types of banks’ efficiency. Equity-financing is of importance for banks because this method gives banks more strength to suffer from financial crisis. Interestingly, the relationship between the quality of the loan and banks' efficiency differs in these two types of banks. CBs may be more prone to issuing riskier loans and bank efficiency is affected by allocating resources towards managing non-performing loans and potential defaults. Loans are typically asset-based and follow Shariah principles, which emphasise risk-sharing and adherence to ethical standards in IBs.

In terms of the adverse impacts caused by GDP, this may be explained by government intervention. Sometimes, central banks implement monetary policies that could lead to lower interest rates and reduced loan quotas. Finally, the impact of inflation was significantly negative at 1% level for IBs but is not significant for CBs. This can be explained by unexpected inflation that cannot be adjusted in a timely manner by the manager, so the revenue failed to cover the cost [4].

### 4.4 Moderating effect of CG

While GDP, CPI, and other macro indices influence the impact of the EU on bank efficiency, CG was also found to be one of the influential factors. Financial institutions’ operations are often assumed to be highly influenced by the regulatory environment, where the governance is significantly different between countries. In this regard, the performance of financial institutions is generally connected with low-quality governance.

[55] found that CG is positively correlated with banks’ efficiency. For instance, to create a peaceful atmosphere for units, CG creates and implements sound laws and regulations, advances democracy, and reduce poverty [4]. also noted that good governance ensures the enforcement of the rule of law and preserves its effectiveness. This benefits banks by reducing the unpredictability and risk associated with starting a business.

Information asymmetric outcomes are also alleviated by good institutional quality, where banks would suffer fewer loan problems [56]. [15] examined the moderating role of CG on the impact of EU on bank lending and concluded that high-quality institutions mitigate the negative impacts of uncertainty on banks and increase credit supply [11]. examined the moderating role of CG on the impact of EU on financial stability and concluded that the adverse impact of uncertainty on the stability of banks can be eased by good governance.

Following the previous study of [11,15], CG was chosen as the moderating variable to form a more accurate conclusion on the nexus between EU and bank efficiency. Table 9 shows the results when adding the moderating role of CG. The significant and positive sign of interaction between CG and the EU in terms of IBs and CBs suggested that good institutional quality can amplify the positive effect of uncertainty on bank efficiency.

**Table 9:** Moderating effect of Governance in CB and IB.

	CB			IB		
	ols	fe	re	ols	fe	re
constant	2.271***	65.047***	3.312***	−1.814	68.869***	5.891
	(0.73)	(6.43)	(0.99)	(2.00)	(9.95)	(3.66)
InAsset	0.119***	0.346***	0.123***	−0.095**	0.373***	−0.065
	(0.02)	(0.07)	(0.03)	(0.04)	(0.11)	(0.07)
CPI	−1.469***	−0.099	−1.557***	−0.222***	−0.352***	−0.385***
	(0.10)	(0.19)	(0.10)	(0.08)	(0.09)	(0.08)
GDP	0.006	−2.792***	−0.024	0.064	−2.904***	−0.231*
	(0.02)	(0.29)	(0.04)	(0.07)	(0.41)	(0.14)
quality	−0.001	−0.000	−0.004	−0.011***	−0.003	−0.006***
	(0.00)	(0.00)	(0.00)	(0.00)	(0.00)	(0.00)
capital	−0.011***	0.018***	−0.006**	−0.003	0.019***	0.000
	(0.00)	(0.00)	(0.00)	(0.00)	(0.01)	(0.00)
wui	1.615***	2.092***	1.793***	0.544	2.382***	1.777***
	(0.27)	(0.29)	(0.28)	(0.61)	(0.60)	(0.60)
CG	−0.186**	0.194	−0.144	0.601***	1.580***	0.530***
	(0.08)	(0.28)	(0.10)	(0.13)	(0.57)	(0.19)
wui*CG	1.212**	1.219**	1.107**	2.056***	3.481***	3.244***
	(0.48)	(0.53)	(0.50)	(0.69)	(0.66)	(0.66)
N	3980	3980	3980	1228	1228	1228
r2	0.096	0.094		0.168	0.101	
r2_a	0.094	0.016		0.162	0.006	
F	52.558***	47.487***		30.689***	15.612***	
BP LM	chibar2(01) = 137.67***			chibar2(01) = 203.17***		
chi2			330.231***			115.107***
Hausman		chi2(8) = 159.84***			chi2(8) = 73.73***	

Standard errors in parentheses.

*p < 0.1, **p < 0.05, ***p < 0.01.

The following can explain the conclusion. First, political stability is one dimension of CG. Operating in a more stable government can amplify the positive effect of uncertainty on bank efficiency. Second, regulatory quality is also a dimension of CG. The positive moderating effect may result from the transparency in financial transactions, regulatory processes and the professional handling of the bureaucracy led by the regulation. Finally, government effectiveness refers to the quality of a government's policies and the competence of its institutions in formulating and implementing effective measures. This index is one dimension of CG. Hence, an effective and responsive government is better equipped to implement appropriate policies to support the banking sector.

### 4.5 Robust test

#### 4.5.1 Replace independent variable

An alternative proxy of the EU was utilised in Table 10. Following the method of [10], the average of the first two quarters of the WUI (proxied by WUJ) was taken as the new independent variable. The results in Table 10 still revealed a positive relationship between EU and both types of banks’ efficiency.

**Table 10:** Robust test1(replace independent variable).

	CB			IB		
	ols	fe	re	ols	fe	re
constant	2.492***	57.709***	7.431***	−5.927***	47.495***	1.959
	(0.72)	(5.06)	(1.74)	(2.02)	(8.90)	(3.66)
InAsset	0.118***	0.178***	0.154***	0.063	−0.004	−0.037
	(0.02)	(0.02)	(0.02)	(0.04)	(0.05)	(0.05)
CPI	−1.528***	−0.181	−1.640***	−0.560***	−0.463***	−0.517***
	(0.10)	(0.18)	(0.11)	(0.08)	(0.08)	(0.08)
GDP	0.011	−2.403***	−0.197***	0.190***	−1.886***	−0.097
	(0.02)	(0.22)	(0.08)	(0.07)	(0.35)	(0.14)
quality	−0.001	0.007***	0.003	−0.012***	−0.007***	−0.008***
	(0.00)	(0.00)	(0.00)	(0.00)	(0.00)	(0.00)
capital	−0.011***	0.009***	0.003	−0.006**	0.016***	0.011***
	(0.00)	(0.00)	(0.00)	(0.00)	(0.00)	(0.00)
wuj	3.677***	4.775***	4.362***	−0.252	3.348**	1.130
	(0.52)	(0.59)	(0.58)	(1.37)	(1.40)	(1.36)
N	3980	3980	3980	1228	1228	1228
r2	0.097	0.120		0.102	0.090	
r2_a	0.096	0.116		0.097	0.076	
F	71.236***	89.987***		23.015***	19.971***	
BP LM	chibar2(01) = 543.85***			chibar2(01) = 1233.40***		
chi2			417.377***			77.514***
Hausman		chi2(6) = 141.26***			chi2(6) = 63.67***	

Standard errors in parentheses.

*p < 0.1, **p < 0.05, ***p < 0.01.

#### 4.5.2 Remove extreme values

Following the method of [57], in order to remove the bias caused by extreme values, the values of 1% and 99% were substituted with data that were less than 1% and greater than 99%, respectively. The regression results are shown in Table 11. The results confirmed the basic conclusion after eliminating extremes.

**Table 11:** Robust test2 (Remove extreme values).

	CB			IB		
	ols	fe	re	ols	fe	re
constant	2.288***	59.874***	3.208***	−5.445***	52.118***	−0.735
	(0.72)	(5.68)	(0.94)	(1.96)	(9.07)	(3.45)
InAsset	0.118***	0.332***	0.123***	0.062*	0.376***	0.097
	(0.02)	(0.07)	(0.02)	(0.04)	(0.11)	(0.06)
CPI	−1.460***	−0.123	−1.547***	−0.527***	−0.332***	−0.486***
	(0.10)	(0.18)	(0.10)	(0.08)	(0.08)	(0.08)
GDP	0.007	−2.586***	−0.020	0.170**	−2.296***	−0.053
	(0.02)	(0.26)	(0.03)	(0.07)	(0.38)	(0.13)
quality	−0.000	0.000	−0.003	−0.011***	−0.005*	−0.007***
	(0.00)	(0.00)	(0.00)	(0.00)	(0.00)	(0.00)
capital	−0.010***	0.018***	−0.006**	−0.006**	0.020***	−0.003
	(0.00)	(0.00)	(0.00)	(0.00)	(0.01)	(0.00)
wui	1.101***	1.617***	1.345***	−0.796*	0.627	−0.114
	(0.18)	(0.21)	(0.19)	(0.47)	(0.49)	(0.47)
N	3980	3980	3980	1228	1228	1228
r2	0.095	0.094		0.107	0.072	
r2_a	0.093	0.016		0.102	−0.024	
F	69.232***	63.142***		24.264***	14.346***	
BP LM	chibar2(01) = 142.41***			chibar2(01) = 292.99***		
chi2			330.294***			60.326***
Hausman		chi2(6) = 155.24***			chi2(6) = 70.58***	

Standard errors in parentheses.

*p < 0.1, **p < 0.05, ***p < 0.01.

### 4.6 Global financial crisis

The global financial crisis (GFC) has led to serious outcomes in many countries' financial systems and is significantly correlated with banks' performance. Hence, an analysis was conducted on the relationship between EU and banks’ efficiency in both pre-GFC, GFC, and post-GFC periods to gain a comprehensive understanding of the findings. Based on the studies of [15,58,59], the sample in this study was divided into three groups, namely the pre-GFC period (2006–2007), GFC period (2008–2009), and post-GFC period (2010–2021). The regression result of this section can be seen in Table 12.

**Table 12:** Regression in terms of GFC.

	Pre-GFC						GFC						Post-GFC					
	CB			IB			CB			IB			CB			IB		
	ols	fe	re	ols	fe	re	ols	fe	re	ols	fe	re	ols	fe	re	ols	fe	re
constant	−0.405	215.968***	2.957	−9.591	35.615	−9.591	−3.231	−33.845	−5.575*	−11.177	31.543	−5.320	−3.497***	57.949***	0.174	−8.784***	46.121***	−2.934
	(1.67)	(47.25)	(2.57)	(8.95)	(152.77)	(8.95)	(2.54)	(34.60)	(3.09)	(8.31)	(83.33)	(14.99)	(0.95)	(7.60)	(2.05)	(2.24)	(11.59)	(4.06)
InAsset	0.009	−0.015	0.004	−0.002	−0.083	−0.002	0.035*	0.009	0.023	0.052	−0.261***	−0.189**	0.155***	0.198***	0.178***	0.071*	−0.007	−0.035
	(0.03)	(0.03)	(0.03)	(0.13)	(0.16)	(0.13)	(0.02)	(0.02)	(0.02)	(0.08)	(0.09)	(0.09)	(0.02)	(0.02)	(0.02)	(0.04)	(0.06)	(0.05)
CPI	−0.240	0.774	−0.984**	1.800	−11.504**	1.800	−0.003	0.427	0.356	−0.183	−0.626	−0.302	−0.552***	0.804***	−0.960***	−0.420***	−0.307***	−0.361***
	(0.31)	(2.19)	(0.45)	(1.45)	(5.41)	(1.45)	(0.52)	(1.03)	(0.60)	(1.61)	(2.49)	(2.30)	(0.14)	(0.30)	(0.17)	(0.08)	(0.10)	(0.10)
GDP	−0.006	−8.601***	−0.003	−0.021	0.576	−0.021	0.024	1.149	0.058	0.350**	−1.065	0.266	0.032	−2.613***	−0.065	0.270***	−1.860***	0.063
	(0.04)	(2.17)	(0.06)	(0.22)	(6.80)	(0.22)	(0.03)	(1.41)	(0.04)	(0.15)	(3.13)	(0.36)	(0.03)	(0.34)	(0.09)	(0.08)	(0.46)	(0.16)
quality	−0.007**	0.005	−0.008**	0.007	−0.033	0.007	0.000	0.012**	0.001	0.033**	0.019*	0.021**	0.000	0.008***	0.006**	−0.011***	−0.007***	−0.008***
	(0.00)	(0.01)	(0.00)	(0.02)	(0.02)	(0.02)	(0.00)	(0.01)	(0.00)	(0.01)	(0.01)	(0.01)	(0.00)	(0.00)	(0.00)	(0.00)	(0.00)	(0.00)
capital	−0.001	−0.000	0.000	0.017**	0.010	0.017**	0.013***	0.013***	0.013***	0.017***	0.006	0.008*	−0.009***	0.010***	0.004	−0.009***	0.016***	0.010**
	(0.00)	(0.00)	(0.00)	(0.01)	(0.01)	(0.01)	(0.00)	(0.00)	(0.00)	(0.01)	(0.00)	(0.00)	(0.00)	(0.00)	(0.00)	(0.00)	(0.00)	(0.00)
wui	−0.557	−1.801	−1.319**	−0.925	−7.929*	−0.925	−0.063	−0.237	−0.070	−1.723	−0.420	−0.580	1.267***	2.279***	1.994***	−0.599	0.593	−0.063
	(0.42)	(1.12)	(0.55)	(2.56)	(4.13)	(2.56)	(0.27)	(0.39)	(0.29)	(1.14)	(0.95)	(0.96)	(0.20)	(0.25)	(0.25)	(0.52)	(0.57)	(0.54)
N	282	282	282	44	44	44	340	340	340	73	73	73	3358	3358	3358	1111	1111	1111
r2	0.027	0.252		0.187	0.418		0.053	0.104		0.223	0.254		0.053	0.071		0.103	0.056	
r2_a	0.006	0.198		0.056	0.106		0.036	0.051		0.152	0.040		0.051	0.066		0.098	0.040	
F	1.295	14.715***		1.422	3.350**		3.125***	6.176***		3.155***	3.171***		31.217***	42.427***		21.186***	10.784***	
BP LM	chibar2(01) = 8.82***			chibar2(01) = 0			chibar2(01) = 8.95***			chibar2(01) = 2.74**			chibar2(01) = 310.01***			chibar2(01) = 885.02***		
chi2			14.228**			8.531			21.413***			16.767**			183.253***			38.728***
Hausman		chi2(6) = 66.03***			chi2(6) = 19.06**			chi2(6) = 36.26***			chi2(6) = 10.30			chi2(6) = 90.27***			chi2(6) = 47.56***	

Standard errors in parentheses.

*p < 0.1, **p < 0.05, ***p < 0.01.

The post-GFC period of banks’ efficiency was found to be positively related with EU, and the coefficient of CBs was greater than that of IBs, which is similar to the basic results. This result is partly consistent with [20], who concluded that GFC has an adverse effect on CBs and has the opposite effect on IBs. However, the sign was not significant during the GFC period. This phenomenon may result from the following reasons: on one hand, the impact of EU on bank efficiency may not have been immediate and could have had time lags, especially during the GFC period. The effects of such uncertainty may have materialised after the GFC or over a more extended period. On the other hand, customer behaviour and preferences may have evolved after the GFC, influencing banks' operations and product offerings. Hence, banks that adapted to these changes and aligned their services with customer needs gained higher efficiency.

## 5 Conclusion

By using data from sample countries, this study empirically examined the impact of EU on the efficiency of two types of banks. Empirical research around the globe tended to focus on cross-country studies within the Gulf Cooperation Council countries or Organization of Islamic Conference countries.

To explore the subjected relationship, empirical analyses using OLS, FEM and REM were conducted. The empirical results revealed that bank efficiency measured by the DEA score was positively related to EU. This demonstrated that the effective anti-risk management actions that the banks took worked. Additionally, CG moderated the positive effect of EU on bank efficiency. The results were robust when the independent variable was replaced and extreme values were removed. Finally, the results were consistent in the post-GFC period.

The results of this study have important policy ramifications. CG was found to positively moderate the impact of EU on banks' efficiency. Therefore, governments and regulatory bodies should prioritise good governance practices, transparency, and effective regulations to create a conducive environment for banks to thrive. Meanwhile, banks should continue to invest in and improve their risk management strategies and mechanisms to ensure financial stability, especially in times of uncertainty. Effective risk management such as adjusting risk management strategies and stress tests can also enhance banks' efficiency and probability during uncertain economic conditions.

However, a key limitation of this research lies in the potential omitted variable biases, generalisability concerns, and endogeneity issues. It should be mentioned that cultural dimensions [60] and political connections [61] can be important factors that can influence banks’ efficiency. Granger causality tests, instrumental variables, and Generalised Method of Moments can also be used to deal with endogeneity issues.

Future research can alternate measures of uncertainty such as oil price variation, standard deviation in real GDP growth (general EU) [15,24], stock market volatility [26], geopolitical risk [62], exchange rate volatility [63], and trade policy uncertainty [64]. Future research can also explore the moderating role of bank regulatory [35] and monetary policy [61] on the EU-bank-efficiency nexus.

## Data availability statement

Data will be made available on request.

## CRediT authorship contribution statement

**Heng Luo:** Writing – review & editing, Writing – original draft, Visualization, Software, Resources, Project administration, Methodology, Investigation, Formal analysis, Data curation, Conceptualization. **Fakarudin Kamarudin:** Validation, Supervision. **Normaziah Mohd Nor:** Validation, Supervision.

## Funding statement

This research did not receive any specific grant from funding agencies in the public, commercial, or not-for-profit sectors.

## Declaration of Competing interest

The authors declare that they have no known competing financial interests or personal relationships that could have appeared to influence the work reported in this paper.
